# Supplementary material for: A tachykinin-like neuroendocrine signalling axis couples central serotonin action and nutrient sensing with peripheral lipid metabolism
Source: Nat Commun. 2017 Jan 27;8:14237. doi: 10.1038/ncomms14237 (PMC5290170; doi:10.1038/ncomms14237)
Supplement: Supplementary Information — Supplementary Figures, Supplementary Tables. [file ncomms14237-s1.pdf]

Supplementary Figure 1

a

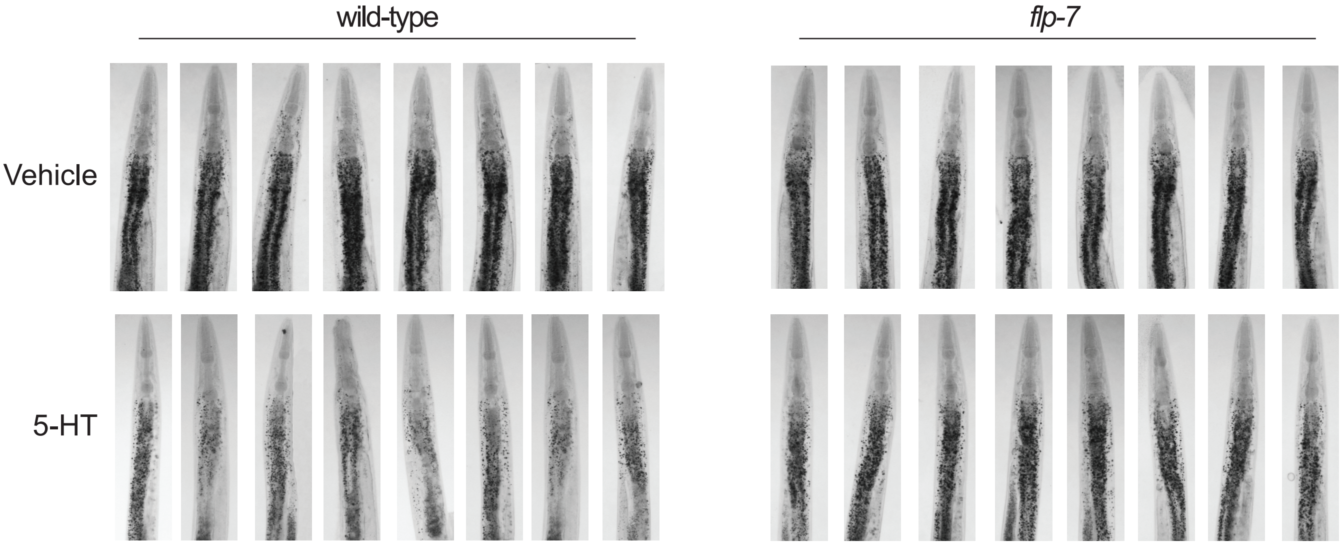

b

FLP-7 (*C. elegans*): S P M Q R S S **M V R F G K R**

Substance P (*H. sapiens*): R P K P Q Q **F** F **G L M** G K R

c

MLGSRFLLLALGLLVLVLAEESAEQQVQEPT*ELEKSGEQLSEEDLIDEQKR**TPMQRSSMVRF**GR**SPMQRSSMVRF**GKR**SPMQRSSMVRF**GKR**SPMQRSSMVRF**GKR**SPMERSAMVRF**GR**SPMDRSKMVRF**GRSSIDRASMVRLGKR**TPMQRSSMVRF**GKRSMEFEMQSNEKNIEDSE

Supplementary Figure 1. The *C. elegans* FLP-7 peptides bear homology to the mammalian tachykinin 'brain-gut' peptides.

(a) Vehicle- and 5-HT-treated wild-type animals and *flp-7* mutant animals fixed and stained with oil Red O. Animals are oriented facing upwards with the pharynx at the anterior end. Images depict the range of the observed phenotypes.

(b) Amino acid sequences for *C. elegans* FLP-7 and *H. sapiens* Substance P are shown. Single dots indicate sequence similarity and double dots indicate sequence identity.

(c) Amino acid sequence of the FLP-7 pro-protein. Bold sequences indicate FLP-7 active peptides cleaved via the action of the pro-protein convertase EGL-3 (Husson and Schoofs, 2007). The star indicates a terminal stop codon in the *flp-7(ok2625)* mutant, thus *flp-7* mutants lack all FLP-7 peptides.

Supplementary Figure 2

Pflp-7::flp-7::GFP

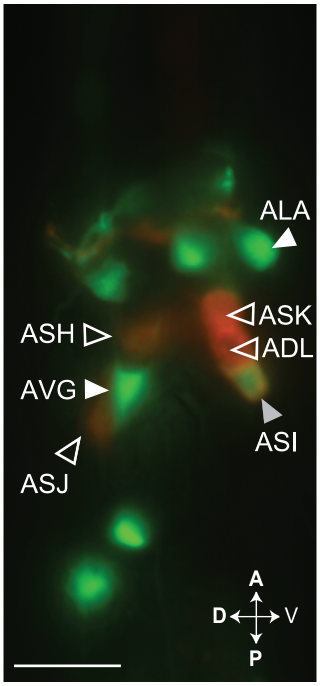

Supplementary Figure 2. FLP-7 expression pattern.

Fluorescent image of a transgenic animal bearing a *flp-7::GFP* transgene (green) with DiI staining (red). We observed robust and clear co-localization of DiI staining and the *flp-7* transgene in the ASI neuron. A, anterior; P, posterior; V, ventral; D, dorsal. Scale bar, 10μm.

Supplementary Figure 3

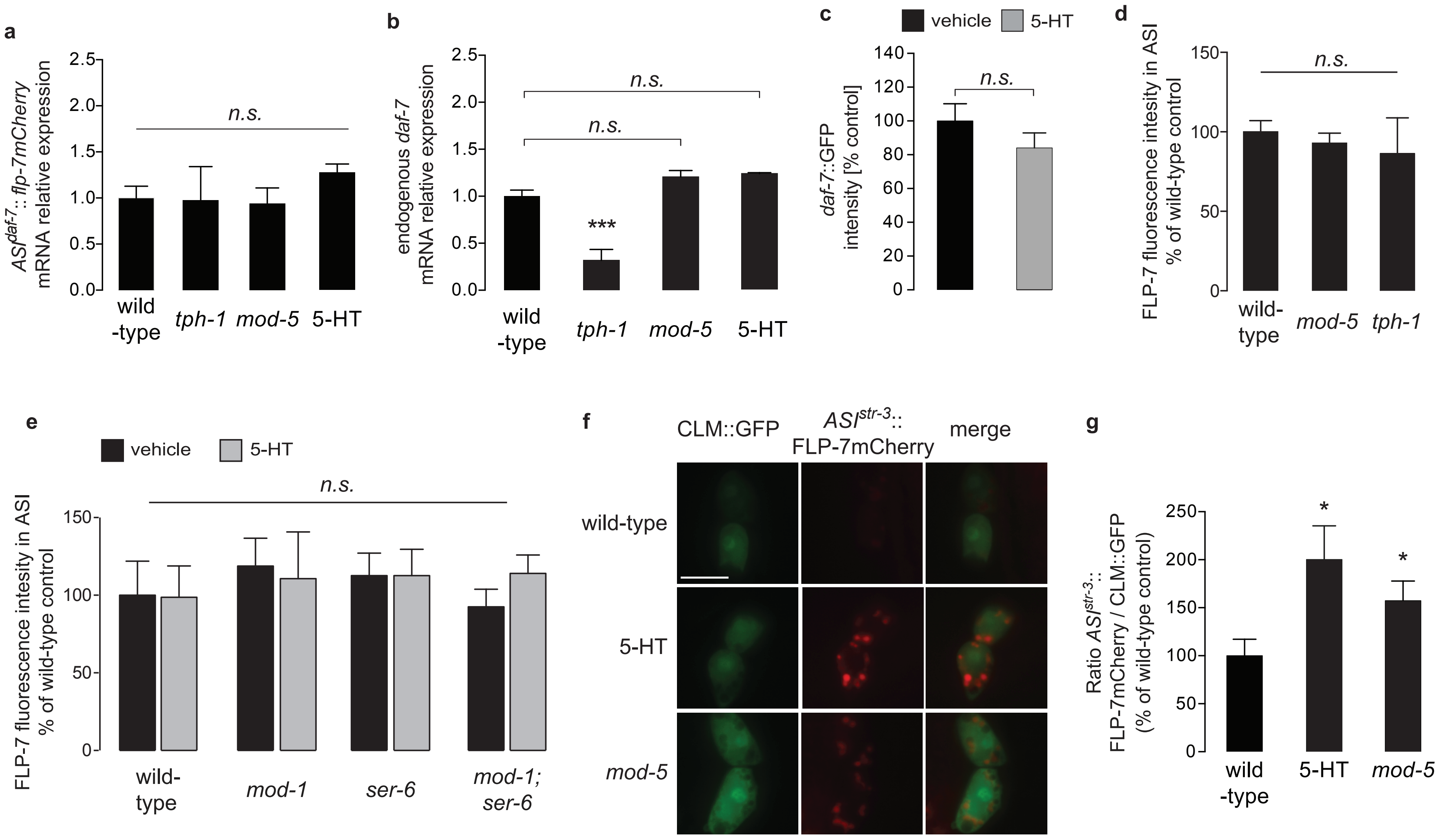

Supplementary Figure 3. Controls to ensure fidelity of FLP-7 secretion.

(a) *FLP-7mCherry* mRNA levels were measured by quantitative PCR in the indicated genotypes and conditions. *ns*, not significant by Student's t-test and one way ANOVA, n=2-3 biological replicates.

(b) Endogenous *daf-7* mRNA levels were measured by quantitative PCR in the indicated genotypes and conditions. ***, $p<0.001$ by one way ANOVA, *ns*, not significant by one way ANOVA and Student t test, n=3 biological replicates.

(c) The *daf-7::GFP* reporter line was treated with vehicle and 5-HT. The fluorescence intensity of *daf-7* expression in the ASI neurons was quantified and expressed as a percentage of vehicle-treated wild-type animals ± SEM (n=18-21). *ns*, not significant by Student's t-test.

(d) Fluorescence intensity of FLP-7 in the ASI neurons was measured in wild-type, *mod-5* and *tph-1* ± SEM (n=12-17) mutants as marked, and expressed as percentage of wild-type animals. *ns*, not significant by Student's t test.

(e) Fluorescence intensity of FLP-7 in the ASI neurons was measured in vehicle- and 5-HT-treated wild-type, *mod-1*, *ser-6* and *mod-1;ser-6* double mutants, and expressed as percentage of vehicle-treated wild-type animals ± SEM (n=12-16). *ns*, not significant by two way ANOVA.

(f) Representative images of vehicle and 5-HT-treated wild-type animals and *mod-5* mutant animals bearing the FLP-7mCherry and CLM::GFP transgenes under the control of *str-3* (ASI) promoter. Left panels, GFP expression in coelomocytes; center panels, secreted FLP-7mCherry uptake in coelomocytes; right panels (merge). Scale bar, 8μm.

(g) An independent ASI promoter, P*str-3* was used to measure FLP-7mCherry secretion. For vehicle- and 5-HT-treated animals bearing FLP-7mCherry and CLM::GFP transgenes, the intensity of FLP-7mCherry fluorescence within a single coelomocyte was quantified and normalized to the area of CLM::GFP expression. Genotypes are indicated

in the figure. Data are expressed as a percentage of the normalized FLP-7mCherry fluorescence intensity of vehicle-treated wild-type animals ± SEM (n=10-17 animals). *, $p<0.05$ by Student's t-test.

Supplementary Figure 4

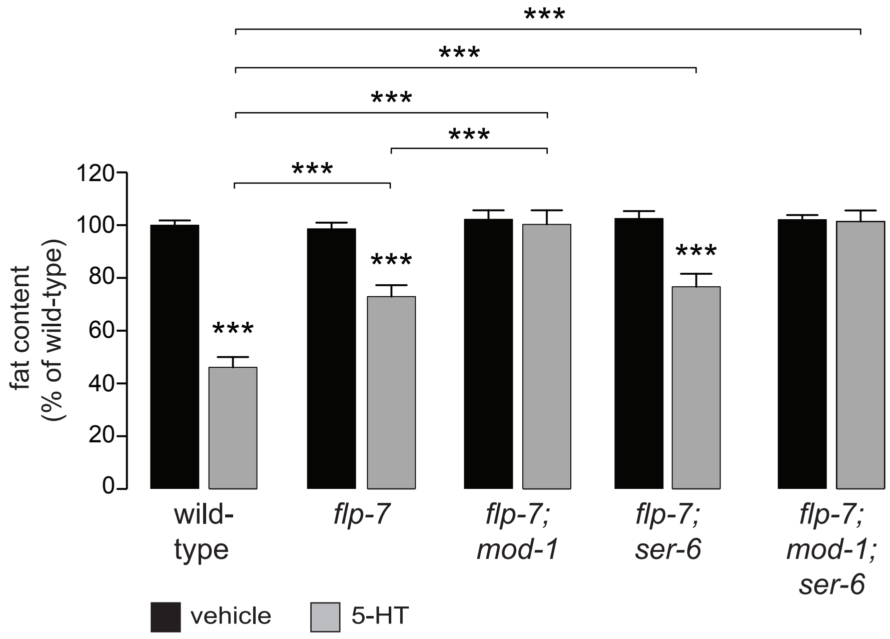

Supplementary Figure 4. FLP-7 functions in an integrated pathway with the 5-HTergic MOD-1 channel and the octopaminergic SER-6 GPCR to regulate body fat loss.

Fat content was quantified for each indicated genotype and is expressed as a percentage of vehicle-treated wild-type animals ± SEM (lower panels; n=12-17). ***, $p<0.001$ by two way ANOVA. Black bars, vehicle treatment, gray bars, 5-HT treatment.

Supplementary Figure 5

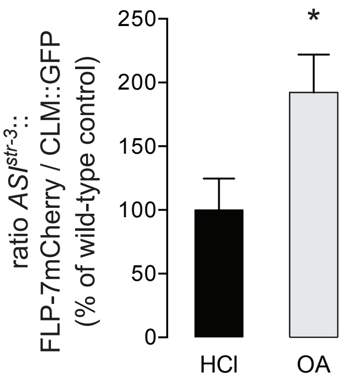

Supplementary Figure 5. FLP-7 is required for OA-induced fat loss.

Animals bearing a FLP-7mCherry under the control of an independent ASI promoter *str-3*, and CLM::GFP transgenes were treated with either vehicle or OA. The intensity of FLP-7mCherry fluorescence was quantified and normalized to the area of the CLM::GFP. Data are expressed as a percentage of the normalized FLP-7mCherry fluorescence intensity of vehicle-treated wild-type animals ± SEM (n=8-12). *, $p<0.05$ by Student's t-test.

Supplementary Figure 6

```
NPR-22 (C.elegans):  MDEGGGIGSSLLSRITTTASEIMMRNEPTTTENPAVQEMNHIYHLTPSMKMLCILFYSILCVCCVYGNVL
TACR2 (H.sapiens):   M-----------------GTCDIVTEANISSGPESNTTGITAFSMPSWQLALWATAYLALVLVAVTGNAI

NPR-22 (C.elegans):  VILVIVYFKRLRTATNILILNLAVADLLISVFCIPFSYWQVLIYDDQRWLFGSMMCSLLAFLQAMAVFLS
TACR2 (H.sapiens):   VIWIILAHRRMRTVTNYFIVNLALADLCMAAFNAAFNFVYASH---NIWYFGRAFCYFQNLFPITAMFVS

NPR-22 (C.elegans):  AWTLVVISFDRWMAIMFLLTPNIRITRRRALYLVAATWIFSILMALPLLFTTRFFEDQDGLPNCGENWTY
TACR2 (H.sapiens):   IYSMTAIAADRYMAIVHPFQPRLSA--PSTKAVIAGIWLVALALASPQCFYSTVTMDQGATKCVVA---W

NPR-22 (C.elegans):  FGDSGEQVRKVYSSMVLILQYVVPQAVLIITYTHIGIKMWNSRVPGMQNGATKKMIVDRHESVKKLVPMV
TACR2 (H.sapiens):   PEDSGGKTLLLYHLVVIALIYFLPLAVMFVAYSVIGLTLWRRAVPGHQAH---GANLRHLQAKKFVKTM

NPR-22 (C.elegans):  ILISALFALCWLPLLILINVIPEFYPDINSWGYILYLWWFAHGLAMSHSMVNPIIYFIRNARFREGFCFF
TACR2 (H.sapiens):   VLVVLTFAICWLPYHLYFILGSFQED-IYCHKFIQQVYLALFWLAMSSTMYNPIIYCCLNHRFRSGFRLA

NPR-22 (C.elegans):  SSKLLPCISFKELRLLTDNTSRSFRNRSRFSGVINPTSSDEKPATSLTRYS---RSGVLDRQTCR--SAR
TACR2 (H.sapiens):   FRCCPWVTPTKEDKLELTPTTS---------LSTRVNRCHTKETLFMAGDTAPSEATSGEAGRPQDGSGL

NPR-22 (C.elegans):  FFEARPL------VVVRNNSANSLA
TACR2 (H.sapiens):   WFGYGLLAPTKTHVEI---------
```

Supplementary Figure 6. Homology between *C. elegans* NPR-22 and *H. sapiens* tachykinin 2 receptor TacR2/NK2R.

Amino acid sequences for *C. elegans* NPR-22 and *H. sapiens* TacR2/NK2R are shown (33% identity, blast score 1e-37). Single dots indicate sequence similarity and double dots indicate sequence identity.

Supplementary Figure 7

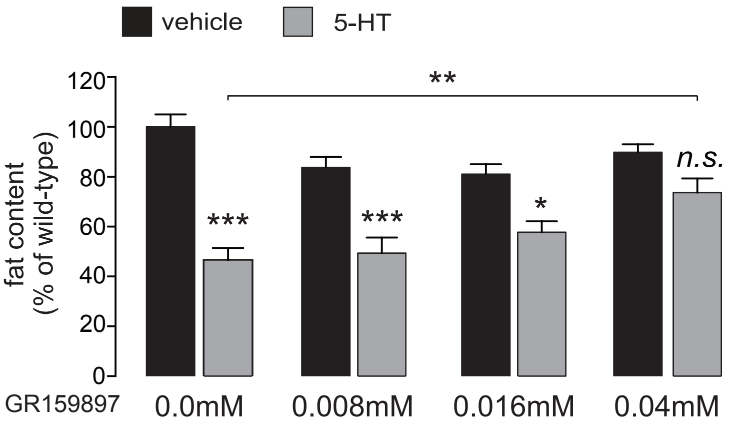

Supplementary Figure 7. Dose-dependent response to TacR2/NK2R antagonist GR 159897.

Wild-type animals were grown on plates containing either vehicle (10% DMSO) or the NK2R antagonist GR159897 at the indicated concentrations. At the completion of development (late L4 stage), animals were transferred to plates containing either vehicle (10% DMSO) or GR159897 at the indicated concentration and either vehicle (HCl) or 5-HT. Fat content was quantified for each condition and is expressed as a percentage of vehicle-treated animals ± SEM (12-19). *, $p<0.05$, **, $p<0.01$ and ***, $p<0.001$ by two way ANOVA.

Supplementary Table 1

Strain	Genotype
MT8004	*unc-13(n2813)*
KQ35	*unc-31(ft1)*
SSR990	*flp-7(ok2625)*
SSR986	*npr-22(ok1598)*
VC40013	*frpr-3(gk240031)*
RB754	*aak-2(ok524)*
WBM697	*crtc-1(tm2069)*
SSR1333	*aak-2(ok524);crtc-1(tm2069)*
SSR1118	*flp-7(ok2625);mod-1(ok103)*
SSR1119	*flp-7(ok2625);ser-6(tm2146)*
SSR1120	*flp-7(ok2625);mod-1(ok103);ser-6(tm2146)*
SSR 647	*N2; ssrIs496[Patgl-1::GFP]*
SSR1171	*flp-7(ok2625); ssrIs496[Patgl-1::GFP]*
SSR1028	*flp-7(ok2625); ssrEx825[Pflp-7:;flp-7::GFP]; ssrEx615[Punc-122::GFP]*
SSR1032	*npr-22(ok1589); ssrEx812[Pnpr-22:npr22::GFP]; ssrEx615[Punc-122::GFP]*
SSR1034	*npr-22(ok1598); ssrEx844[Pges-1::npr-22::GFP]; ssrEx615[Punc-122::GFP]*
SSR1037	*npr-22(ok1598); ssrEx891[Punc-31::npr-22::GFP]; ssrEx615[Punc-122::GFP]*
SSR1089	*N2; ssrEx884Pdaf-7::flp-7::GFP sense]; ssrEx885Pdaf-7::flp-7::mCherry antisense]; ssrEx615[Punc-122::GFP]*
SSR1100	*N2; ssrIs[Pdaf-7::GFP]; ssrEx886[Pflp-7::flp-7::mCherry]; ssrEx615[Punc-122::GFP]*
SSR1113	*flp-7(ok2625); ssrEx920[Pdaf-7::flp-7::mCherry]; ssrEx429[Pmyo-3::mCherry]*
SSR1135	*flp-7(ok2625);npr-22(ok1598); ssrEx920[Pdaf-7::flp-7::mCherry]; ssrEx844[Pges-1::npr-22::GFP]*
SSR1141	*flp-7(ok2625);npr-22(ok1598); ssrEx920[Pdaf-7::flp-7::mCherry]*
SSR1142	*flp-7(ok2625);npr-22(ok1598); ssrEx920[Pges-1::npr-22::GFP]*
SSR1164	*N2; ssrIs919[Pdaf-7::flp-7mCherry]; ssrIs615[Punc-122::GFP]*
SSR1173	*npr-22(ok1598); ssrIs496[Patgl-1::GFP]*
SSR1175	*mod-5(n3314); ssrIs919[Pdaf-7::flp-7mCherry]; ssrIs615[Punc-122::GFP]*
SSR1176	*ser-6(tm2146); ssrIs919[Pdaf-7::flp-7mCherry]; ssrIs615[Punc-122::GFP]*
SSR1179	*mod-1(ok103);ser-6(tm2146); ssrIs919[Pdaf-7::flp-7mCherry]; ssrIs615[Punc-122::GFP]*
SSR1181	*tph-1(mg280); ssrIs919[Pdaf-7::flp-7mCherry]; ssrIs615[Punc-122::GFP]*
SSR1212	*N2; ssrEx787[Pflp-7::flp-7::GFP]*
SSR1240	*unc-31(ft1); ssrIs919[Pdaf-7::flp-7mCherry]; ssrIs615[Punc-122::GFP]*
SSR1241	*mod-1(ok103); ssrIs919[Pdaf-7::flp-7mCherry]; ssrIs615[Punc-122::GFP]*
SSR1298	*N2; ssrEx1005[Pstr-3::flp-7mCherry]; ssrEx615[Punc-122::GFP]*
SSR1302	*flp-7(ok2625); ssrEx1006[Pstr-3::flp-7::GFP]*
SSR1326	*aak-2(ok524); ssrIs919[Pdaf-7::flp-7mCherry]; ssrIs615[Punc-122::GFP]*
SSR1328	*aak-2(ok524); ssrEx1019[Pgpa-4::aak-2::GFP]*
SSR1331	*flp-7(ok2625);npr-22(ok1598); ssrIs496[Patgl-1::GFP]*
SSR1332	*npr-22(ok1589); ssrEx787[Pflp-7::flp-7::GFP]*
SSR1335	*aak-2(ok524);crtc-1(tm2069); ssrEx1020[Pgpa-4::crtc-1::GFP}; ssrIs919[Pdaf-7::flp-7mCherry]; ssrIs615[Punc-122::GFP]*
SSR1340	*N2; ssrEx1021[Pgpa-4::crtc-1CA::GFP]; ssrIs919[Pdaf-7::flp-7mCherry]; ssrIs615[Punc-122::GFP]*
SSR1337	*mod-5(n3314); ssrEx1006[Pstr-3:flp-7::mCherry]*
SSR1338	*aak-2(ok524);crtc-1(tm2069); ssrIs919[Pdaf-7::flp-7mCherry]; ssrIs615[Punc-122::GFP]*

Supplementary Table 2

No.	Gene Name	Gene ID	Forward Primer Sequence*	Reverse Primer Sequence*
**Promoter Cloning Primers**				
1	*flp-7*	F49E10	GGG GAC AAC TTT GTA TAG AAA AGT TGC TTT CTG ATT CTT GTT GC	GGG GAC TGC TTT TTT GTA CAA ACT TGT CAT TTC TAA AAG TCT TTG AAT G
2	*str-3*	M7.13	GGG GAC AAC TTT GTA TAG AAA AGT TGT GGT GAA GAT TTG TTC AAG GAC G	GGG GAC TGC TTT TTT GTA CAA ACT TGT CAT TCT AGA GTT CCT TTT GAA ATT GAG GC
3	*gpa-4*	T07A9	GGG GAC AAC TTT GTA TAG AAA AGT TGC GTC ATC ATG GGA TAA CTA CAA TC	GGG GAC TGC TTT TTT GTA CAA ACT TGT CAT GTG TTC ACA AAA TGA ATA AGT GGC
4	*npr 22*	Y56H11AL	GGG GAC AAC TTT GTA TAG AAA AGT TGC GTT TTT CGA GGG AGT GC	GGG GAC TGC TTT TTT GTA CAA ACT TGT CAT ATT TGC TCA CGA ATT AGA TGA C
5	*unc-31*	ZK897	GGG GAC AAC TTT GTA TAG AAA AGT TGG AGG TGG TCT GTA TGT GAC GG	GGG GAC TGC TTT TTT GTA CAA ACT TGT CAT GAT GTT CCA AAC GAA GAC TGC
**cDNA Cloning Primers**				
1	*flp-7*	F49E10	GGG GAC AAG TTT GTA CAA AAA AGC AGG CTT GCT TGG ATC CCG CTT CC	GGG GAC CAC TTT GTA CAA GAA AGC TGG GTA TTC GCT GTC CTC GAT GTT CTT C
2	*flp-7* sense	F49E10	GGG GAC AAG TTT GTA CAA AAA AGC AGG CTC AAG AAC CAA CTG AGT GAG TTG AG	GGG GAC CAC TTT GTA CAA GAA AGC TGG GTA GTT TTC CGA GTC TGA ACT TCA GC
3	*flp-7* antisense	F49E10	GGG GAC AAG TTT GTA CAA AAA AGC AGG CTG TTT TCC GAG TCT GAA CTT CAG C	GGG GAC CAC TTT GTA CAA GAA AGC TGG GTA CAA GAA CCA ACT GAG TGA GTT GAG
4	*npr-22*	Y56H11AL	GGG GAC AAG TTT GTA CAA AAA AGC AGG CTT GGA CGA AGG AGG G	GGG GAC CAC TTT GTA CAA GAA AGC TGG GTA AGT ACA TGG TAG ATC AAT TTT CTT AAT G
**qPCR Primers**				
1	*flp-7*mCherry	*NA*	GTT TTG GCA AAC GTT CGA TGG A	TGA AGC GCA TGA ACT CCT TGA
2	*daf-7*	B0412	GAG TAC CTT AAG AAC GAA ATT CTC GAC C	CCC TAT ACA TCT CCA GGT AGA CTG AC

* Gateway specific sequence is underlined for each primer
